# Supplementary material for: Near field optical visualization of the nanoscale phase percolation dynamics of a VO2 oscillator
Source: Nat Commun. 2026 Jan 14;17:600. doi: 10.1038/s41467-026-68300-y (PMC12808705; doi:10.1038/s41467-026-68300-y)
Supplement: Supplementary file 1 — Supplementary Information [file 41467_2026_68300_MOESM1_ESM.pdf]

Supplementary Information

Near field optical visualization of the nanoscale phase percolation dynamics of a VO_2 oscillator

Kajal Tiwari[1, †], Zhong Wang[1, †], Yishen Xie[1], Ajesh Kollakuzhiyil Gopi[1], Jae-Chun Jeon[1], Ke Xiao[1,*], and Stuart S. P. Parkin[1,*]

[1]Max Planck Institute for Microstructure Physics, Weinberg 2, 06120 Halle (Saale), Germany

[†]These authors contributed equally to this work

*Corresponding authors: ke.xiao@mpi-halle.mpg.de, stuart.parkin@mpi-halle.mpg.de

Supplementary Note 1- Film characterization

XRD *θ-2θ* scan of a typical pulsed laser deposition (PLD) grown, ~10 nm thick, VO_2 thin film on a TiO_2(001) single crystalline substrate. The XRD pattern exhibits a peak at 2θ = 65.9° near the substrate TiO_2 (002) peak at 2θ = 62.73°, which corresponds to the (002) crystal surface of the metallic tetragonal phase of VO_2, as shown in the Supplementary Figure 1a [1]. An atomic force microscopy (AFM) image of a typical VO_2 thin film, shown in the Supplementary Figure 1b, exhibits a smooth surface. The root mean square (RMS) roughness was ~0.1 nm. Details on the growth of the films can be found in the methods section.

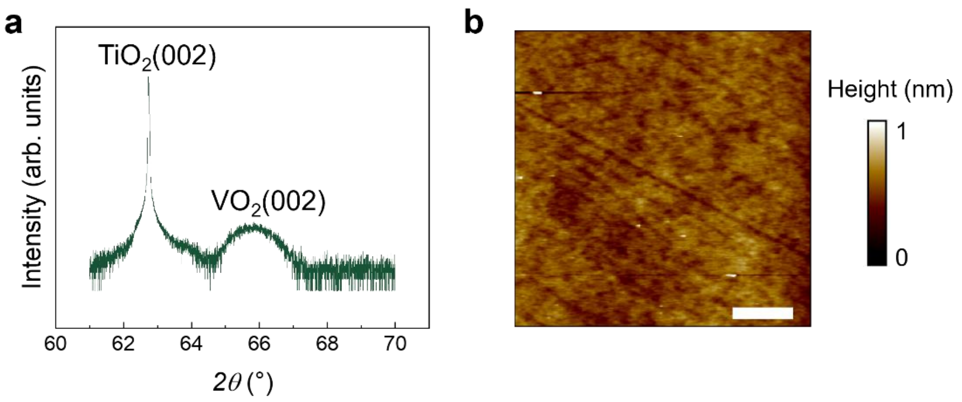

Supplementary Figure 1: VO_2 film characterization. **a**, X-ray diffraction (XRD) *θ-2θ* curve showing the growth of an epitaxial VO_2 film oriented along (001) on a TiO_2(001) single crystalline substrate. **b**, AFM topography image of the pristine surface of a ~10 nm thick VO_2 film. Scale bar: 2 µm.

Supplementary Note 2- Temperature dependent insulator-to-metal phase transition in VO_2

To determine the degree of metallicity of the percolation pathways emerging during the current-induced Insulator-to-Metal Transition (IMT), we compare the s-SNOM signal strength from these pathways with that observed in the metallic phase during the temperature-driven IMT without applied current. In Supplementary Figure 2, we present a sequence of s-SNOM images of the IMT

in VO_2 obtained at successive temperatures as the temperature is swept from 300 K to 310 K in the absence of applied current. Our findings align with previous reports[2] and reveal a percolative metallic network that progressively increases in extent with temperature. These patches eventually coalesce, leading to a more homogeneous metallic state. The transition begins above 300 K. Note that 310 K is the upper temperature limit of our s-SNOM cryostat.

Next, we perform a comparative analysis of the s-SNOM signals from percolative regions during both the temperature-dependent and current-induced IMT. The latter are shown in the main text in Fig. 1c. s-SNOM signal normalization is crucial for an accurate comparison of images, as s-SNOM signal can be significantly affected by variations in optical alignment due to changes in sample temperature, tip replacement, or adjustments in tip parameters such as amplitude and setpoint. Here we normalize the s-SNOM signal strength to that measured from the middle of one of the Au electrodes. The normalized s-SNOM amplitude of the metallic patches during the temperature dependent IMT is 0.34 which is similar in strength to that from the isotropic region observed during the current-induced IMT at 295 K (see main text, Fig. 1c, right).

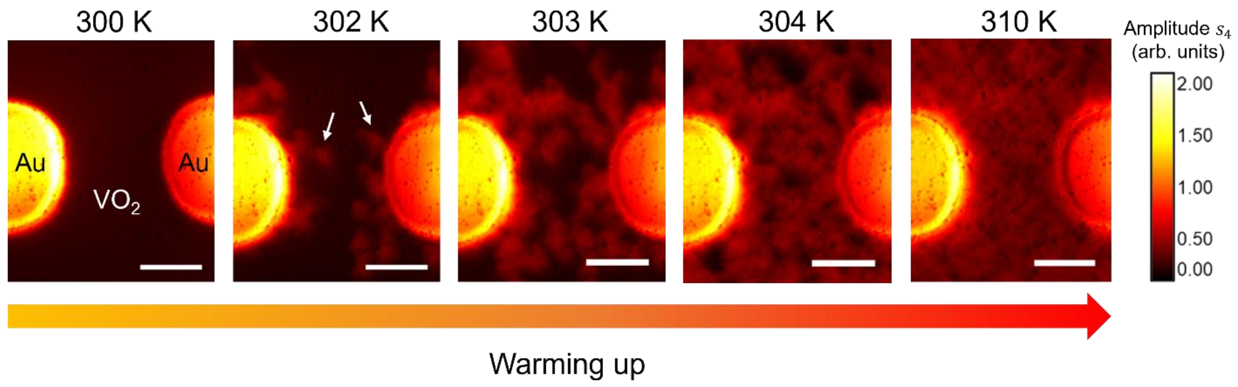

Supplementary Figure 2: Temperature dependent insulator to metal transition in VO_2. 4th order tip-demodulated s-SNOM amplitude (s_4) images obtained at successive stages of the IMT in VO_2 during a warming-up process, showing the emergence of high reflectivity metallic patches (a few indicated by white arrows) at intermediate stages in the absence of applied current. Scale bar: 2 µm.

Supplementary Note 3- Current induced IMT at 291 K

We performed s-SNOM imaging of the current-induced IMT in device 2 at 291 K, as shown in Supplementary Figure 3a under applied current $I \geq I_{(th)}$ and Supplementary Figure 3b after switching off the applied current. This temperature lies between the temperatures T_1 and T_2 of the main text. In Supplementary Figure 3a, a filamentary feature and metallic regions are clearly visible in the 2nd order tip-demodulated s-SNOM phase image (φ_2). The 2nd or 3rd order tip-demodulated s-SNOM signals inherently minimize background scattering from the tip-shaft and illuminated areas. Higher-order signals provide better spatial resolution and lower background but can have reduced signal to noise ratio (SNR), necessitating careful alignment and optimized scanning parameters at low temperatures. Although measurements in the manuscript primarily use 4th order signals, we occasionally present 2nd or 3rd order data in the Supplementary Information when higher orders have insufficient SNR.

Upon turning the current off (Supplementary Figure 3b), a small portion of the filament remains, more clearly observed in the phase image as compared to the amplitude image. The phase image provides superior contrast for smaller changes in metallicity[3]. These remnant metallic regions influence the threshold current required for switching to the metallic state. While previous reports have attributed the presence of stable multi-resistance states in the hysteresis regime of the transition to the formation of filamentary percolation alone, our results reveal a scenario consisting of both filaments and metallic patches.

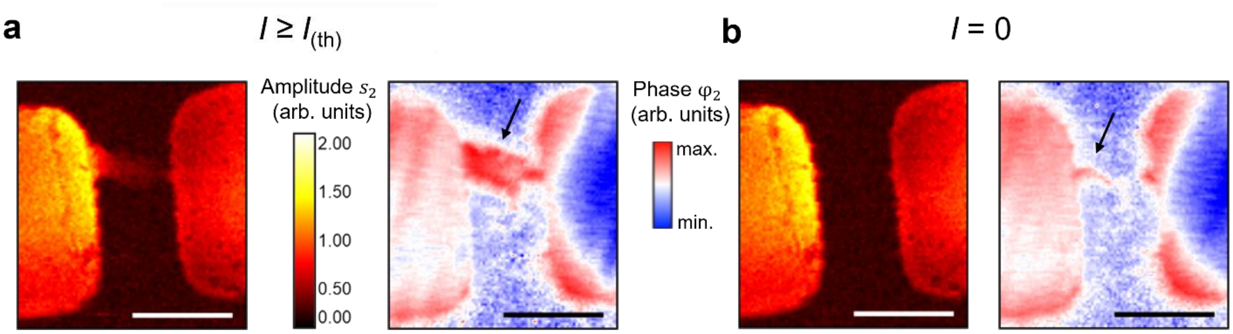

Supplementary Figure 3: Current induced IMT at 291 K. **a,** 2nd order tip-demodulated s-SNOM amplitude (s_2) and phase (φ_2) images of device 2 acquired at 291 K under a constant applied current $I \geq I_{(th)}$, where $I_{(th)}$ denotes the threshold current required to induce IMT. The device enters a low-resistance state following the formation of a filament-like structure, indicated by the black arrow. Scale bar: 2 µm. **b,** s-SNOM amplitude (s_2) and phase (φ_2) images taken after switching off the current. The device reverts to an insulating state; however, residual percolation paths, marked by black arrow, result in a modified resistance. This change is reversible upon cooling the film below the transition temperature and re-warming it to 291 K. The same color scale is applied to subfigure b. Scale bar: 2 µm.

Supplementary Note 4- Persistent metallic patch (PeMP) in various devices

The persistent metallic patch (PeMP) has been observed for multiple devices at 285 K in Supplementary Figure 4. Devices 1 and 2 have been used for most of the data presented in the main text. Device 4 has been used for Kelvin Probe Force Microscopy (KPFM) measurements discussed in Supplementary note 10. Devices 4 and 5 have larger PeMPs due to an excessive heating from a current that was applied well above the threshold current. For all the devices the formation of a PeMP region was key to the observation of a current induced oscillatory state.

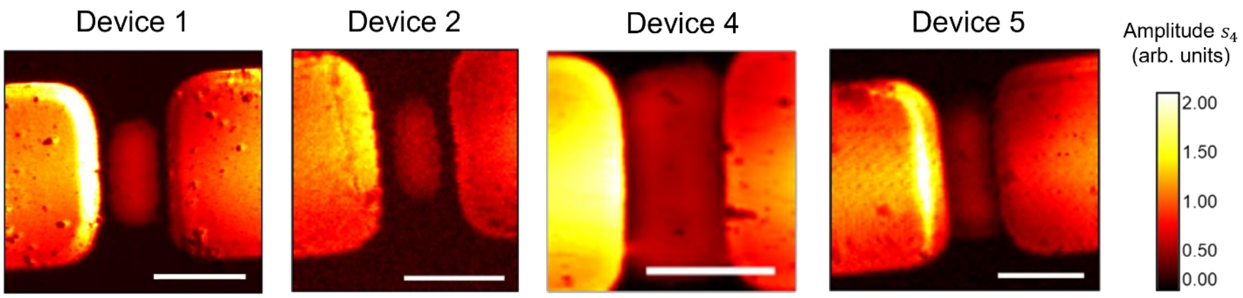

Supplementary Figure 4: Formation of persistent metallic patch (PeMP) in various devices at 285 K. 4th order tip-demodulated s-SNOM amplitude (s_4) images of 4 × 2 µm^2 VO_2 devices at 285 K in the absence of applied current. The same color scale is applied to all the images. Scale bar: 2 µm.

Supplementary Note 5- Filament width

We estimated the widths of the filaments formed in the metallic state at 285 K using the full width at half maximum (FWHM) of a Lorentzian that was fitted to the s-SNOM amplitude linecuts. The narrowest filament was ~ 136 nm wide in a device of dimension 2 × 2 µm^2. The s-SNOM amplitude curve of the linecut is presented in Supplementary Figure 5a, with the inset showing the s-SNOM phase image. The filaments observed in Device 1 are shown in Supplementary Figure 5b, where the inset highlights filaments number 1 and 2 along with their respective linecuts. Filaments 1 and 2 have widths of 204 nm and 226 nm, respectively.

In Supplementary Figure 5c, we present a line-cut across the transient filament formed during the oscillatory state of device 1 at 285 K, with the inset corresponding to Fig. 2d of the manuscript. The maximum signal of 0.4 at the transient filament is consistent with the maximum signal observed in Fig. 2g.

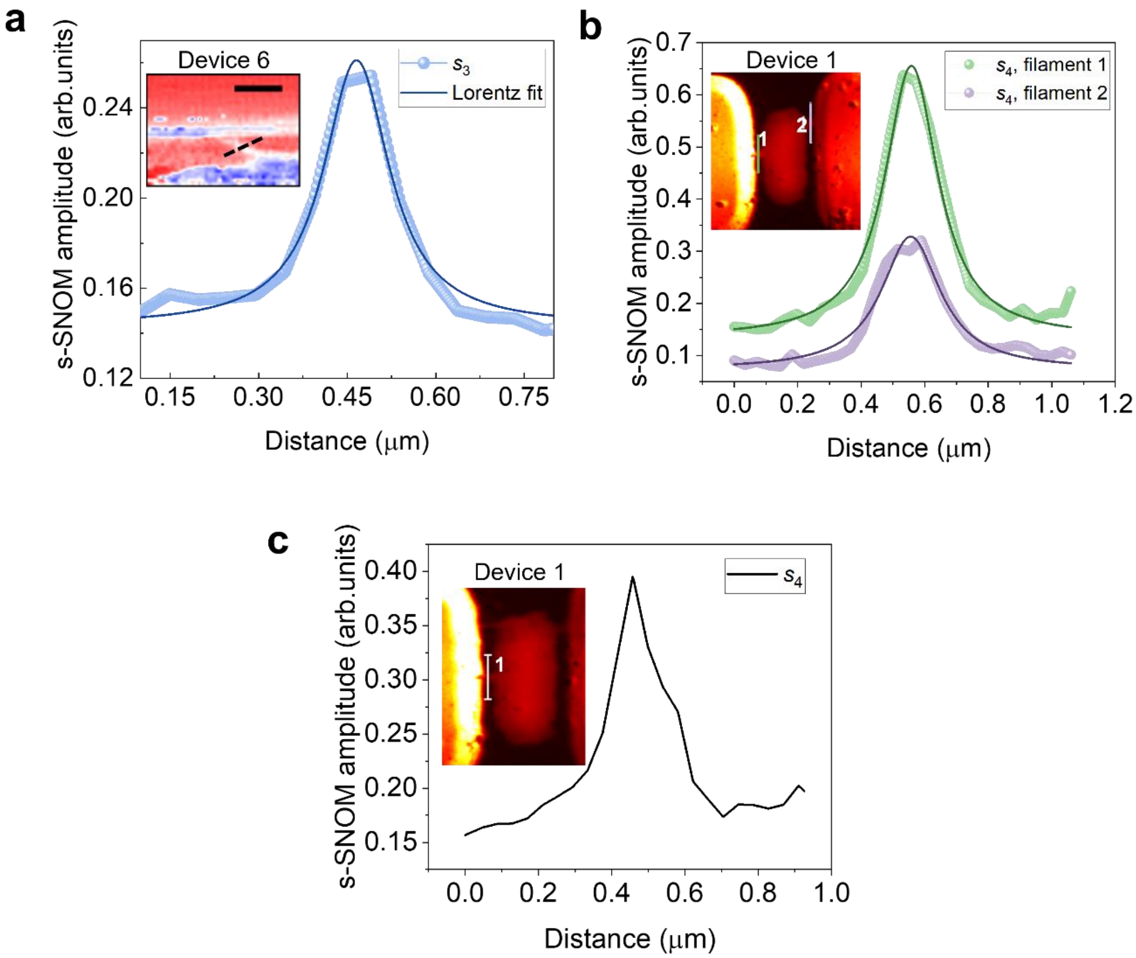

Supplementary Figure 5: Estimation of filament width. a, 3rd order tip-demodulated s-SNOM amplitude (s_3) signal for a device with a size of 2 × 2 μm^2 along the line-cut (black dashed line) shown in the inset s-SNOM phase (φ_3) image. Scale bar: 500 nm. **b,** 4th order tip-demodulated s-SNOM amplitude (s_4) curves for the two filaments (1 & 2) in device 1 (4 × 2 μm^2) along the line-cuts shown in the inset s-SNOM amplitude (s_4) image (see Fig. 2b of the main text). **c,** s-SNOM amplitude linecut at the transient filament location shown in the inset (see Fig. 2d of the main text). The maximum signal of the transient filament is 0.4 (arb. units), consistent with the maximum signal observed in Fig. 2g of the main text.

Supplementary Note 6- *V-I* and *R-I* curves in the oscillatory regime

The initial application of a high current at a given temperature leads to a persistent modification in the electrical transport properties, as evident from the exemplary voltage and resistance versus current curves at 280 K shown in Supplementary Figure 6 for a device with dimensions of 2 × 2 μm^2. For three successive current sweeps, the device exhibits a progression through resistance states each lower than the prior. The most interesting observation is the large drop in resistance that is observed after the 1st loop.

The transport measurements indicate the presence of non-volatile changes in the device triggered by the current-induced IMT. Our s-SNOM imaging reveals the formation of a PeMP if a sufficiently high current is applied.

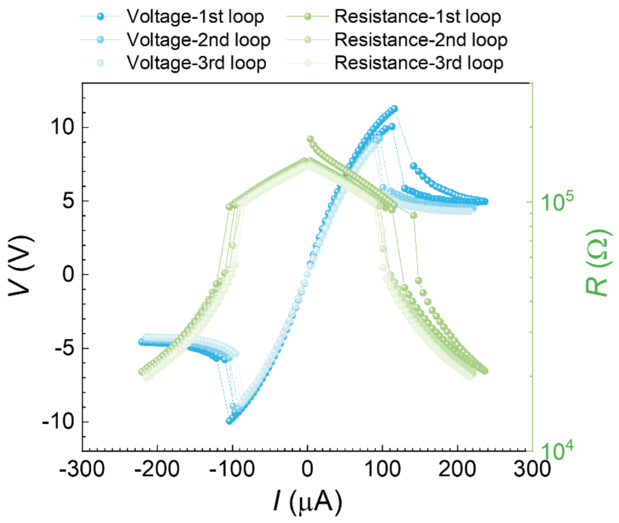

Supplementary Figure 6: *V-I* and *R-I* curves. *V-I* and *R-I* curves of a 2 × 2 μm^2 device indicate the presence of non-volatile changes during successive loops of current sweeps from 0 to values exceeding $I_{(th)}$.

Supplementary Note 7- PeMP shrinking and reappearance

Temperature dependent s-SNOM imaging of the PeMP in the absence of an applied current reveals a gradual shrinking of the PeMP during cooldown, as illustrated in Supplementary Figure 7 for

three temperatures: 285 K, 200 K, and 100 K. The image at 100 K shows no PeMP. Upon warming, the PeMP re-emerges, as observed in the s-SNOM image at 290 K. The microscopic mechanism underlying PeMP formation is discussed in the main text (Fig. 3a), where energy-dispersive X-ray microscopy (EDX) is employed to show an oxygen deficiency within the PeMP region.

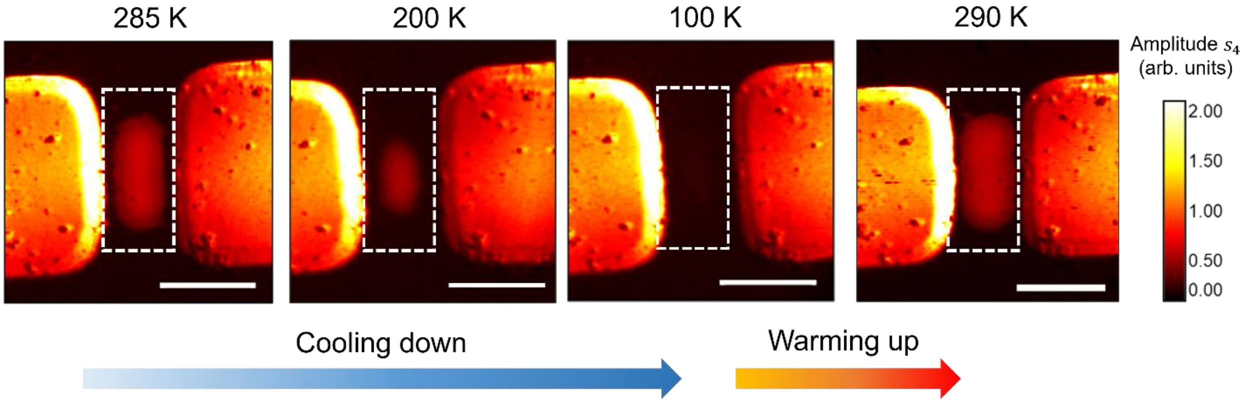

Supplementary Figure 7: PeMP shrinking and reappearance. 4th order tip-demodulated s-SNOM amplitude (s_4) images of device 1 (4×2 μm^2) at successive stages of cooldown showing a shrunk PeMP enclosed by a white dashed box. PeMP reappears on warming up, as shown in the image at 290 K. The same color scale is applied to all the images. Scale bar: 2 μm.

Supplementary Note 8- PeMP pulsation in time

During current induced resistance oscillations, we observe a broadening of the PeMP in the s-SNOM images. This raises the question of whether the PeMP undergoes a dynamic pulsation, expanding and contracting as a result of thermal cycling during each oscillation period, such that the apparent broadening represents a time-averaged outcome of the image integration. Our results show that a complex interplay between PeMP pulsation and filamentary formation and disappearance underlies the observed oscillatory behavior.

To investigate potential PeMP movement, we conducted s-SNOM imaging under a constant current, denoted as $I_{(osc)}$, just below the onset of stable resistance oscillations. In this regime, resistance oscillations occur intermittently with long intervals of inactivity ranging upto

tens of milliseconds (see Supplementary Figure 8a), providing a suitable time window to resolve non-averaged spatial changes in s-SNOM. The s-SNOM imaging was performed with an integration time of 3.3 ms per pixel. Under these conditions, we observed shifts at the PeMP periphery, as shown in the right panel of Supplementary Figure 8b. The images display different scenarios under the applied current (current on) condition, corresponding to: completely inactive during scaning (no expansion of PeMP), fully active during scanning (expansion of PeMP), and mixture of inactivity and activity during scanning (non-continuous expansion of PeMP), The image size is 40 × 80 px.

Based on our previous observations that the PeMP expands and contracts with temperature (Supplementary Figure 7), we attribute these current induced fluctuations in the periphery of the PeMP to temperature changes induced by the current induced oscillatory dynamics i.e. changes in resistance that will also be associated with small changes in temperature due to changes in resistive heating.

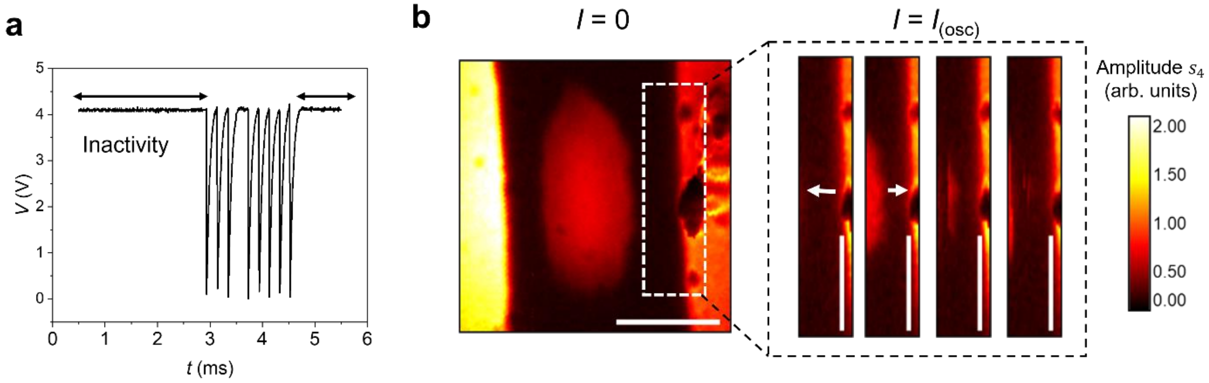

Supplementary Figure 8: PeMP pulsation. a, $V(t)$ output showing oscillations interrupted by intervals of inactivity at constant current applied at the minimal threshold for the oscillatory regime (denoted as $I_{(osc)}$). **b**, 4th order tip-demodulated s-SNOM image (s_4) of device 2 (4 × 2 μm^2) on the left shows the PeMP in the absence of applied current. The series of images on the left capture the PeMP periphery under an applied $I_{(osc)}$. Movement of the PeMP periphery is evident in the zoomed-in regions (indicated by the white arrow), where the periphery shifts closer to or farther from the electrode. The same color scale is applied to all the images. Scale bar: 1 μm.

Supplementary Note 9- Simulation of temperature and current density distribution

A finite-element simulation of the current density and temperature distribution was conducted using COMSOL Multiphysics. The model consisted of a TiO_2 substrate, a 10 nm-thick VO_2 film and two Au contact electrodes (40 nm thick each) on top of the film. The film region within the space between the electrodes (active region) was 4 µm (width) × 2 µm (length), the same size as in most of our devices. The physical equations of the model are:

$$\boldsymbol{J} = -\sigma \nabla V, \quad (1)$$

$$\nabla \cdot \boldsymbol{J} = 0, \quad (2)$$

$$Q_{\text{Joule}} = -\boldsymbol{J} \cdot (\nabla V), \quad (3)$$

$$-\nabla \cdot (\kappa \nabla T) = Q_{\text{Joule}}, \quad (4)$$

where $\boldsymbol{J}$, V, Q_{Joule}, and T are current density, voltage, Joule heat density and temperature, respectively. σ and κ are electrical and thermal conductivities of the material, respectively.

Each simulation was performed in two steps. First, current was applied between the electrodes and the current density distribution in the film was computed. In the second step, Joule heat distribution was computed based on the solution of the first step, and the steady-state heat transfer in the whole model was simulated to give the temperature distribution. The temperature at the bottom of the TiO_2 substrate was set to 285 K. All other surfaces in the model were set to be electrically and thermally insulating. Tetrahedral domain meshes and triangular surface meshes were used for the finite-element method. The experimental electrical conductivity of VO_2 (insulating state) was used for the film. The thermal conductivity of the materials was taken from literature (accessed from the COMSOL database).

The temperature distribution at an applied current 140 µA is presented in Fig. 3c and d of the main text. The simulated current density distribution, as shown in Supplementary Figure 9a, indicates a relatively uniform distribution within the active region along the x axis.

The current distribution along line 2, the orange line in Supplementary Figure 9a, is shown in Supplementary Figure 9c. This reveals a plateau across the active region, indicating a relatively

uniform current density and no dip in the active region near the electrodes. In contrast, the temperature profile presented in the main text (Fig. 3c and 3d) displays a pronounced dip. This temperature distribution closely mirrors the characteristic shape of the PeMP and aligns with the presence of insulating areas between the PeMP and the electrodes, emphasizing the importance of localized thermal effects in its formation.

Materials	Electrical conductivity at 285 K	Thermal conductivity at 285 K ($W \cdot K^{-1} \cdot m^{-1}$)
Au	Ideal conductor	314 [4]
TiO_2	Ideal insulator	8.69 [5]
VO_2	5.0 $S \cdot cm^{-1}$	4.4 [6]

Supplementary Table: Summary of materials properties used in the COMSOL simulation.

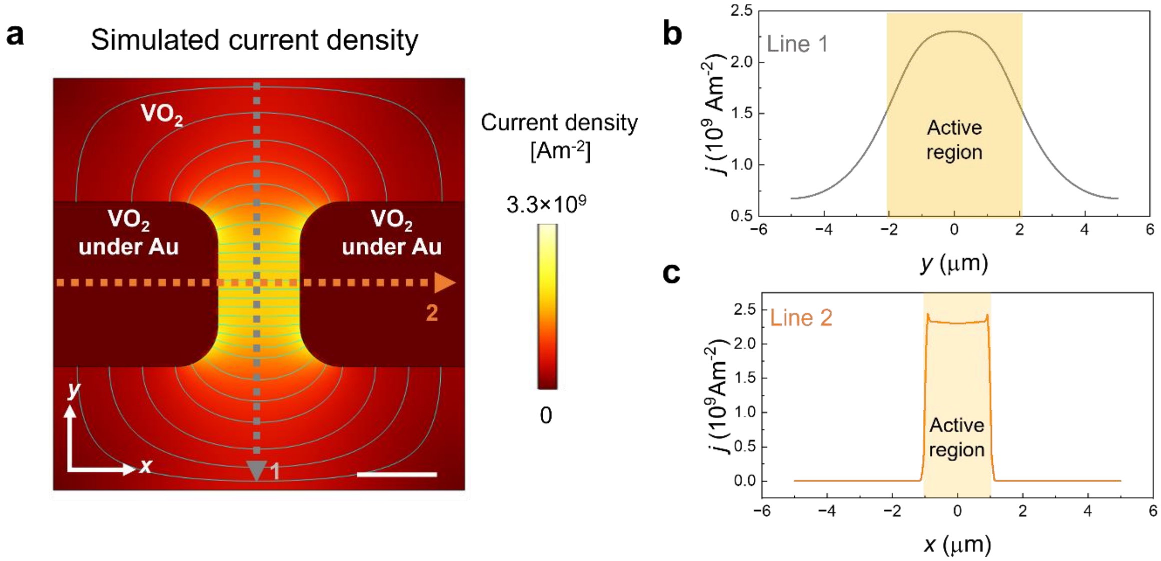

Supplementary Figure 9: Simulation of current density distribution. a, Simulated current density distribution for a 4 × 2 μm² device at applied current 140 μA. Current flow lines (grey) show a uniform distribution in the active region. **b,** Line-cut along y (shown by grey dashed line 1 in **a**). The active region is shaded in yellow. **c,** Line-cut along x (shown by orange dashed line 2

in **a**) shows no dip in the current density near the electrodes. The small peaks at the edges of the active region are an artefact. Scale bar: 2 μm.

Supplementary Note 10- Kelvin Probe Force Microscopy (KPFM)

Kelvin Probe Force Microscopy (KPFM) results at room temperature, shown in Supplementary Figure 10b, alongside the corresponding room temperature AFM topography in Supplementary Figure 10a, are presented for device 4 which hosts a PeMP below IMT. AFM topography has no signatures of any height differences in the active region which already hosts the PeMP, consistent with Fig. 3a and 3b in the main text. In contrast, KPFM, which probes the local surface potential and is sensitive to variations in the work function, reveals a region of elevated surface potential within the active area. Notably, the spatial profile of this high-potential region closely matches the PeMP geometry observed in the s-SNOM amplitude image (see Supplementary Figure 4). The agreement between the KPFM and s-SNOM strongly supports the interpretation that the PeMP exhibits a change in electronic properties compared to the surrounding film.

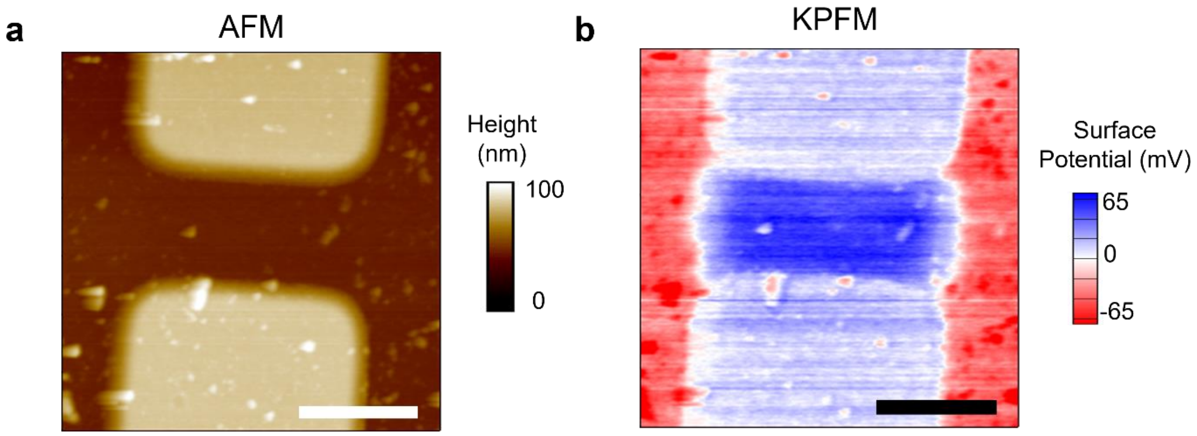

Supplementary Figure 10: Kelvin Probe Force Microscopy (KPFM). a, Room temperature AFM image of device 4 hosting a PeMP below IMT. **b,** Room temperature KPFM reveals a PeMP.

Supplementary Note 11- AFM amplitude and phase

Unlike AFM height, which primarily reflects surface topography, AFM amplitude and phase are sensitive to local variations in stiffness, adhesion, and chemical composition [7, 8]. These channels can therefore reveal subtle features that remain invisible in height images. In Supplementary Figure 11, we compare s-SNOM amplitude with AFM phase, amplitude, and height images of the current-induced IMT at 295 K, 290 K, and 285 K. As stated in the manuscript, AFM height shows no discernible changes after the transition compared to the initial state (Supplementary Figure 11a). At 295 K and 290 K, the metallic regions likewise exhibit no detectable variations in AFM amplitude or phase. At 285 K, where PeMP form and oscillations occur, slight variations emerge in the AFM phase image (Supplementary Figure 11d). Because AFM phase is particularly sensitive to chemical modifications, these observations are consistent with our EDX and KPFM measurements.

IMT in VO_2 involves a monoclinic to rutile structural change accompanied by a ~0.3% contraction along the rutile c-axis [9]. While AFM height differences between insulating and metallic domains have been reported in bulk crystals of similar materials[10], our film is only 10 nm thick. The expected topographic change from a 0.3% contraction is therefore on the order of ~0.03 nm, which lies well below AFM's practical detection limit. This could explain the absence of any measurable height contrast in our data.

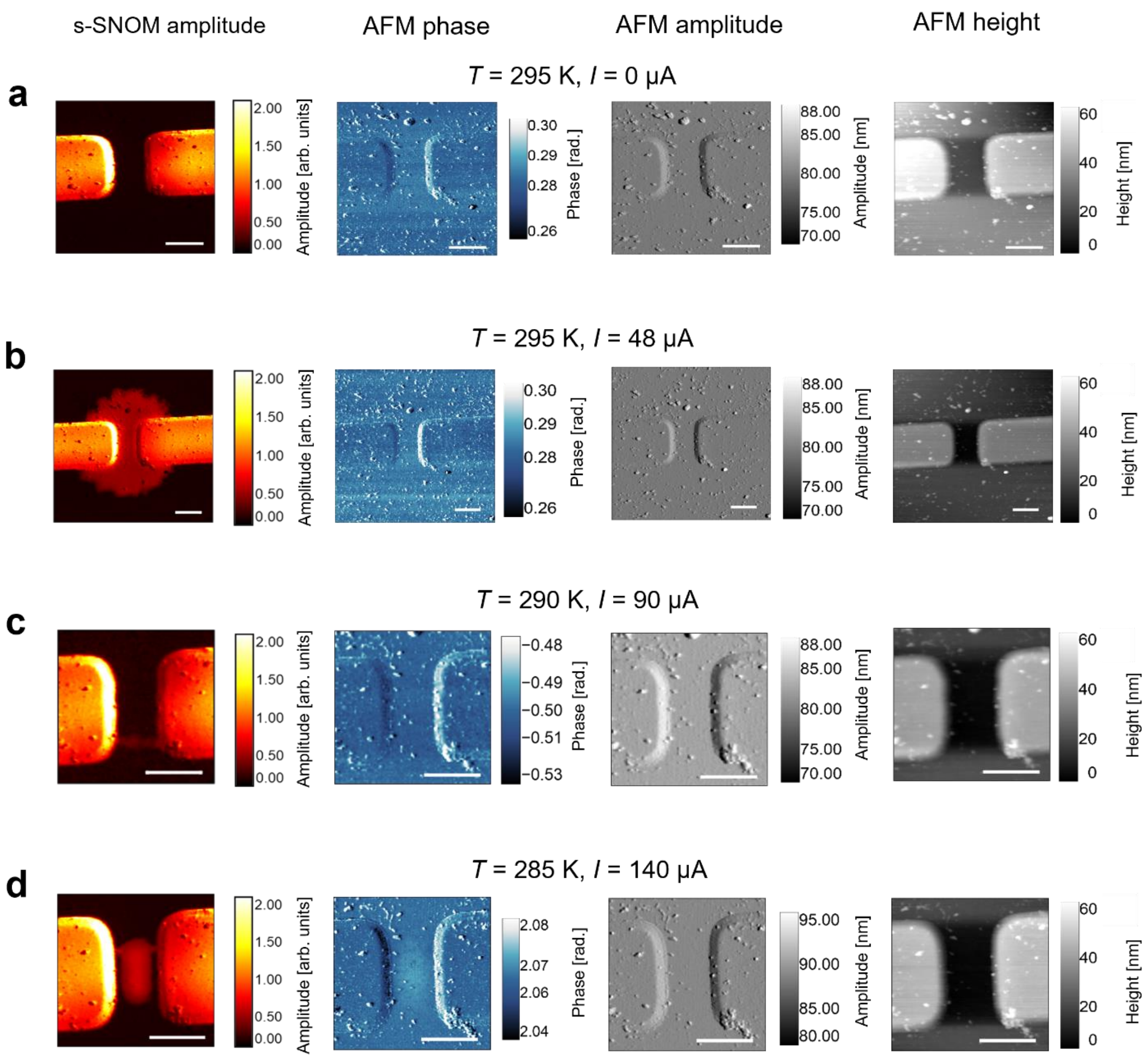

Supplementary Figure 11: s-SNOM amplitude and AFM phase, amplitude and height. s-SNOM amplitude (s_4), AFM phase, amplitude and height for device 1 at **a,** T_1 = 295 K and I = 0, **b,** T_1 = 295 K and I = 48 μA (corresponding to $I_{(th)}$), **c,** T_2 = 290 K and I = 90 μA ($I_{(th)} \geq 80$ μA), **d,** T_3 = 285 K and I = 140 μA ($I_{(th)} \geq 130$ μA). $I_{(th)}$ correspond to the threshold current required to induce the IMT. Scale bar: 2 μm

Supplementary Note 12- Possible scenarios of filament formation

In Fig. 2g of the main text, we show the time evolution of the s-SNOM amplitude at a fixed point (marked by a black dot for filament 1 in the s-SNOM phase image of Fig. 2d) illustrating the stochastic flickering of filaments during unstable oscillations (see Supplementary Figure 8a) at the minimum threshold current for device 1. To provide some intuitive physical scenarios connecting filament flickering to macroscopic resistivity oscillations, one can consider the possibilities as shown in Supplementary Figure 12. As shown in the figure, the s-SNOM signal labeled 1 corresponds to the absence of a filament, as it matches the no-filament signal (~0.15 in Supplementary Figure 5c). Possible scenarios at the probed location (yellow dashed box) include A-F, where stable filaments are indicated by solid orange lines and transient filaments by translucent orange lines. Among these, scenario B is likely the case. Signal 2, which matches the transient filament value (~0.4 in Supplementary Figure 5c), could arise from scenarios G or H, with scenario G, involving transient filaments on both sides, being more consistent. Signal 3 lies at an intermediate level and is a mixture of scenarios of signal 1 and 2. Signal 4 slightly exceeds the transient filament level and is consistent with scenarios I or J, where the probed filament exhibits a more stable character. However, since the fully stable filament signal reaches ~0.65 (see supplementary Figure 5b), this minor increase of ~0.03 is more likely within experimental uncertainty rather than a change in filament behavior. The corresponding device voltage-time traces and the filament state they likely represent are illustrated in the figure.

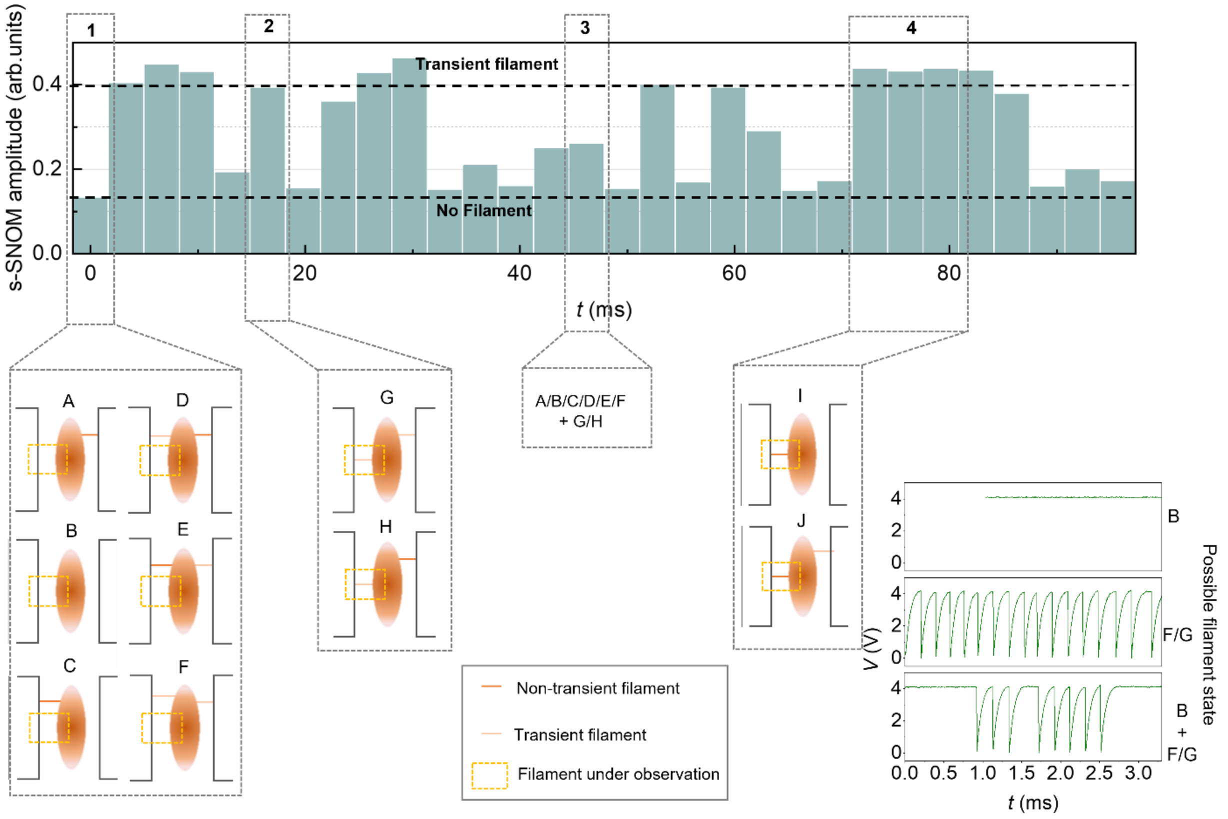

Supplementary Figure 12: Possible scenarios of filament formation. s-SNOM amplitude (s_4) time trace for the point of interest (shown in yellow dashed box) and the corresponding possible scenarios of filament formation and the relation to the $V(t)$ output of the device. Transient filament is denoted by light orange line while stable or non-transient filament is dark orange line

Supplementary Note 13- Laser heating effect on IMT in VO_2

Below we quantify the temperature rise for our mid-IR illumination at λ = 10 µm with incident power $P_{\text{inc}} = 0.8$ mW, both for the near-field (s-SNOM) hotspot (~40 nm, set by the tip apex) and determines the resolution of the s-SNOM system and for a typical diffraction-limited far-field laser spot.

First, we calculated the absorptance of the VO_2 film which determines how much of the incident power is absorbed by the film:

In our setup, the laser light of wavelength $\lambda = 10$ μm is p-polarized and incident at an angle of $\theta_i = 60°$. For a VO_2 film with complex refractive index $n_t = n + ik$ and thickness $d = 10$ nm on top of TiO_2 substrate (considered semi-infinite), ignoring interference effects and considering multiple reflections from air-VO_2 and VO_2-TiO_2 surfaces denoted by Fresnel reflectance R_1 and R_2 [11], absorptance can be written as [12] :

$$A_p = 1 - R_p - T_p = \frac{(1-R_1)(1-\eta)(1+R_2\eta)}{1-R_1R_2\eta^2} \tag{5}$$

Where $\eta = e^{-\frac{\alpha d}{\cos\theta_1}}$ is the one-pass attenuation inside the film with absorption coefficient $\alpha = 4\pi k/\lambda$, transmission angle $\theta_1 = \arcsin\left(\frac{n_i}{n_t}\sin\theta_i\right)$. Using the insulating VO_2 values $n = 2.8$, $k = 0.15$ [13] and TiO_2 values $n = 2.0$, $k = 0.15$ [14], we obtain $A_p \approx 1.4 \times 10^{-3} \approx 0.14$ %.

Rise in temperature by far-field laser spot:

In the far-field, the absorbed power is:

$$P_{abs} = P_{inc}\,A_p = 0.8\times 10^{-3} \times 1.4\times 10^{-3} \approx 1.12 \times 10^{-6}\ \mathrm{W} \tag{6}$$

To estimate the rise in temperature, we need to calculate the spot size of the diffraction limited beam. For λ = 10 μm and a parabolic mirror with NA ≈ 0.46, the diffraction-limited $1/e^2$ diameter is: $D_{FF} \approx \frac{2\lambda}{\pi\,NA} \approx 13.8$ μm so we take a conservative radius $a = \sqrt{2}\sigma = \frac{D_{FF}}{2} \approx 7$ μm. According to the Gaussian heat flux model on a semi-infinite substrate, the absorbed heat flux is [15, 16, 17]:

$$q(r) = \frac{P_{abs}}{2\pi\sigma^2}\, e^{-\frac{r^2}{2\sigma^2}}\ \mathrm{Wm^{-2}} \tag{7}$$

Given the thermal conductivities $\kappa_{VO2} = 4.4$ $Wm^{-1}K^{-1}$ and $\kappa_{TiO2} = 8.69$ $Wm^{-1}K^{-1}$, the steady state peak rise at the substrate surface is [17]:

$$\Delta T_{sub}(0) = \frac{q(0)a}{2\,\kappa_{TiO2}} = \frac{P_{abs}}{2\pi\,\kappa_{TiO2}\,a} = \frac{1.12\times10^{-6}}{2\pi\times8.69\times7\times10^{-6}} \approx 2.9\ \mathrm{mK} \tag{8}$$

Film contribution at the center (added in series) and ignoring the film-substrate interface ($\Delta T_{\text{interface}}$) effect:

$$\Delta T_{\text{film}}(0) = \frac{q(0)\,d}{\kappa_{\text{VO2}}} = \frac{P_{\text{abs}}\,d}{\pi\,\kappa_{\text{VO2}}\,a^2} = \frac{1.12\times10^{-6}\times10\times10^{-9}}{\pi\times4.4\times7\times10^{-6}\times7\times10^{-6}} \approx 0.017\text{ mK} \quad (9)$$

Hence, the total temperature rise is 2.92 mK.

Rise in temperature by near-field nano-spot:

To calculate the absorbed power in the nano-spot, we calculated the fraction of the incident power falling in this nanospot without considering any near-field enhancement effects. For a tiny disk $r \ll a$, area fraction from FF spot (diameter 13.8 µm) to tip (diameter 40 nm, $a = 20$ nm):

$$f = \left(\frac{40\text{ nm}}{13.8\text{ µm}}\right)^2 \approx 8.4\times10^{-6} \quad (10)$$

The absorbed power within the 40 nm disk is $f \times P_{\text{abs}} \sim 0.009$ nW. The Gaussian heat flux model yields a baseline local rise:

$$\Delta T_{sub}(0) = \frac{P_{\text{abs}}}{2\pi\,\kappa_{\text{TiO2}}\,a} = \frac{0.009\times10^{-9}}{2\pi\times8.69\times20\times10^{-9}} \approx 0.008\text{ mK} \quad (11)$$

$$\Delta T_{\text{film}}(0) = \frac{P_{\text{abs}}\,d}{\pi\,\kappa_{\text{VO2}}\,a^2} = \frac{0.009\times10^{-9}\times10\times10^{-9}}{\pi\times4.4\times20\times10^{-9}\times20\times10^{-9}} \approx 0.016\text{ mK} \quad (12)$$

$$\Delta T = \Delta T_{\text{sub}} + \Delta T_{\text{film}} \approx 0.024\text{ mK} \quad (13)$$

However, a field enhancement at the s-SNOM tip apex cannot be ignored. Therefore, we scale the local intensity by a field-enhancement factor F (one of the experimentally reported value for non-resonant tips [18] was 23) [19]. In this intensity-scaling picture,

$$P_{\text{abs}} \approx F\,P_{\text{abs},0}, \Delta T \approx F\,\Delta T_0 \quad (14)$$

For $F = 23$, $\Delta T \approx 0.6$ mK. This value provides an intuitive, field-enhancement based estimate of local heating under the tip.

As an unrealistic upper limit, if we assume that the total power absorbed by the material in the FF spot (1.12×10^{-6} W) can be coupled to the tiny volume fraction ($a = 20$ nm) under the tip due to an extreme field enhancement, the rise in substrate surface temperature would be:

$$\Delta T_{\mathrm{sub}}(0) = \frac{P_{\mathrm{abs}}}{2\pi\, \kappa_{\mathrm{TiO2}}\, a} = \frac{1.12 \times 10^{-6}}{2\pi \times 8.69 \times 20 \times 10^{-9}} \approx 1.03\ \mathrm{K} \tag{15}$$

And the film temperature:

$$\Delta T_{\mathrm{film}}(0) = \frac{P_{\mathrm{abs}}\, d}{\pi\, \kappa_{\mathrm{VO2}}\, a^2} = \frac{1.12 \times 10^{-6} \times 10 \times 10^{-9}}{\pi \times 4.4 \times 20 \times 10^{-9} \times 20 \times 10^{-9}} \approx 2.03\ \mathrm{K} \tag{16}$$

$$\Delta T\ = \Delta \mathrm{T}_{\mathrm{sub}} + \Delta T_{\mathrm{film}} \approx 3.1\ \mathrm{K} \tag{17}$$

An overestimated laser heating induced temperature rise of 3.1 K is still not enough to cause an IMT at 285 K sample temperature when the warming transition is ~308 K. Even at 295 K, the closest to the transition, we still required an electrical bias (≈ 48 µA) to form a conducting element. By contrast, previous reports on laser-heating experiments (at 532 nm, ~500 nm spot, ~100 nm VO_2 films) typically used >10 mW optical power and reported filament loss when the power dropped below ~7.8 mW [20]. At 532 nm, insulating VO_2 has a penetration depth of ~125 nm, so a 100 nm film absorbs a substantial fraction of the beam, whereas in our case the 10 µm mid-IR penetration depth is ~5 µm, and our VO_2 is only 10 nm thick, so only a tiny fraction of the incident mid-IR power is absorbed, yielding negligible heating in the VO_2 film.

Supplementary References

1. Jeong J, Aetukuri N, Graf T, Schladt TD, Samant MG, Parkin SSP. Suppression of Metal-Insulator Transition in VO_2 by Electric Field-Induced Oxygen Vacancy Formation. *Science* 2013, **339**(6126)**:** 1402-1405.

2. Qazilbash MM, Brehm M, Chae BG, Ho PC, Andreev GO, Kim BJ, *et al.* Mott transition in VO_2 revealed by infrared spectroscopy and nano-imaging. *Science* 2007, **318**(5857)**:** 1750-1753.

3. Stiegler JM, Huber AJ, Diedenhofen SL, Rivas JG, Algra RE, Bakkers EPAM, *et al.* Nanoscale Free-Carrier Profiling of Individual Semiconductor Nanowires by Infrared Near-Field Nanoscopy. *Nano Lett* 2010, **10**(4)**:** 1387-1392.

4. Young HD. *University Physics*, 7 edn. Addison Wesley, 1992.

5. Kingery WD, Francl, J., Coble, R. L. & Vasilos, T. . Thermal conductivity: X, data for several pure oxide materials corrected to zero porosity. *Journal of the American Ceramic Society* 1954, **37:** 107-110.

6. Kizuka H, Yagi T, Jia JJ, Yamashita Y, Nakamura S, Taketoshi N, *et al.* Temperature dependence of thermal conductivity of VO_2 thin films across metal-insulator transition. *Jpn J Appl Phys* 2015, **54**(5).

7. Pang GKH, Baba-Kishi KZ, Patel A. Topographic and phase-contrast imaging in atomic force microscopy. *Ultramicroscopy* 2000, **81**(2)**:** 35-40.

8. Howard AJ, Rye RR, Houston JE. Nanomechanical basis for imaging soft materials with tapping mode atomic force microscopy. *J Appl Phys* 1996, **79**(4)**:** 1885-1890.

9. Jeong J, Aetukuri NB, Passarello D, Conradson SD, Samant MG, Parkin SSP. Giant reversible, facet-dependent, structural changes in a correlated-electron insulator induced by ionic liquid gating. *P Natl Acad Sci USA* 2015, **112**(4)**:** 1013-1018.

10. Zhang JW, McLeod AS, Han Q, Chen XZ, Bechtel HA, Yao ZZ, *et al.* Nano-Resolved Current-Induced Insulator-Metal Transition in the Mott Insulator Ca_2RuO_4. *Phys Rev X* 2019, **9**(1).

11. Palik ED. Handbook of Optical-Constants. *J Opt Soc Am A* 1984, **1**(12)**:** 1297-1297.

12. Fox M. *Optical properties of solids*, 2 edn. Oxford university press Inc., Newyork: USA, 2010.

13. Beaini R, Baloukas B, Loquai S, Klemberg-Sapieha JE, Martinu L. Thermochromic VO-$_2$-based smart radiator devices with ultralow refractive index cavities for increased performance. *Sol Energ Mat Sol C* 2020, **205**.

14. Siefke T, Kroker S, Pfeiffer K, Puffky O, Dietrich K, Franta D, *et al.* Materials Pushing the Application Limits of Wire Grid Polarizers further into the Deep Ultraviolet Spectral Range. *Adv Opt Mater* 2016, **4**(11)**:** 1780-1786.

15. Loze MK, Wright CD. Temperature distributions in laser-heated semi-infinite and finite-thickness media with convective surface losses. *Appl Optics* 1998, **37**(28)**:** 6822-6832.

16. Loze MK, Wright CD. Temperature distributions in semi-infinite and finite-thickness media as a result of absorption of laser light. *Appl Optics* 1997, **36**(2)**:** 494-507.

17. Orekhov A, Rabinskiy L, Fedotenkov G. Analytical Model of Heating an Isotropic Half-Space by a Moving Laser Source with a Gaussian Distribution. *Symmetry-Basel* 2022, **14**(4).

18. Atkin JM, Berweger S, Jones AC, Raschke MB. Nano-optical imaging and spectroscopy of order, phases, and domains in complex solids. *Adv Phys* 2012, **61**(6)**:** 745-842.

19. Wagner M, Fei Z, McLeod AS, Rodin AS, Bao WZ, Iwinski EG, *et al.* Ultrafast and Nanoscale Plasmonic Phenomena in Exfoliated Graphene Revealed by Infrared Pump-Probe Nanoscopy. *Nano Lett* 2014, **14**(2)**:** 894-900.

20. Feng C, Li BW, Dong Y, Chen XD, Zheng Y, Wang ZH, *et al.* Quantum imaging of the reconfigurable VO_2 synaptic electronics for neuromorphic computing. *Sci Adv* 2023, **9**(40).
